# Cell Blebbing upon Addition of Cryoprotectants: A Self-Protection Mechanism

**DOI:** 10.1371/journal.pone.0125746

**Published:** 2015-04-13

**Authors:** Renquan Ruan, Lili Zou, Sijie Sun, Jing Liu, Longping Wen, Dayong Gao, Weiping Ding

**Affiliations:** 1 Center for Biomedical Engineering, University of Science and Technology of China, Hefei, Anhui 230027, China; 2 Department of Electronic Science and Technology, University of Science and Technology of China, Hefei, Anhui 230027, China; 3 Department of Laboratory Medicine, University of Washington, Seattle, WA, 98195, United States of America; 4 School of Life Science, University of Science and Technology of China, Hefei, Anhui 230027, China; 5 Department of Mechanical Engineering, University of Washington, Seattle, WA, 98195, United States of America; University of California San Diego, UNITED STATES

## Abstract

In this work, the mechanism of cell bleb formation upon the addition of cryoprotectants (CPAs) was investigated, and the role of cell blebs in protecting cells was determined. The results show that after adding CPAs, the hyperosmotic stress results in the breakage of the cortical cytoskeleton and the detachment of the cell membrane from the cortical cytoskeleton, causing the formation of cell blebs. Multiple blebs decrease the intracellular hydrostatic pressure induced by the extracellular hyperosmotic shock and alleviate the osmotic damage to cells, which reduces the cell mortality rate. In the presence of a low concentration of CPAs, cell blebs can effectively protect cells. In contrast, in the presence of a high concentration of CPAs, the protective effect is limited because of severe disruption in the cortical cytoskeleton. To determine the relationship between blebs and the mortality rate of cells, we defined a bleb index and found that the bleb index of 0.065 can be regarded as a reference value for the safe addition of DMSO to HeLa cells. The bleb index can also explain why the stepwise addition of CPAs is better than the single-step addition of CPAs. Moreover, the mechanism of the autophagy of cells induced by the hyperosmotic stress was studied, and the protective effect associated with the autophagy was compared with the effect of the blebbing. The findings reported here elucidate a self-protection mechanism of cells experiencing the hyperosmotic stress in the presence of CPAs, and they provide significant evidence for cell tolerance in the field of cryopreservation.

## Introduction

Cell blebs are spherical cellular membrane protrusions that inflate and retract on a timescale of minutes, resulting from either the detachment of the cell membrane from the actin cortex [1] or the localized rupture of the actin cortex [2]. Cell blebs attract a great deal of interest because of their dynamic features connected to dramatic cellular reorganization with the roles in cytokinesis [3], cell spreading [4], virus uptake [5, 6], apoptosis [7], and locomotion of tumor and embryonic cells [8, 9]. In addition, increasing evidence points to an essential role for blebs during cell migration in 3-D environments [10–12]. The life cycle of cell blebs is dynamic, and they often rapidly expand, abruptly stop at diameters of a few micrometers, and slowly shrink as the actin cortex is reconstituted under the plasma membrane [13]. Rho-ROCK-myosin has been defined as essential signaling of contractility for the bleb retraction [14, 15].

The formation and expansion of cell blebs are commonly driven by mechanical perturbations, such as micropipette suction [16] and osmotic shock [17]. Cell blebs provide valuable insights into cell mechanics as some interesting biophysical phenomena can be discovered during the life cycle of cell blebs. For example, the change in adhesion energy between the actin cortex and the cell membrane can be investigated by the generation of cell blebs [18], and the stress build-up in the cortex and the mechanical properties of the cortex can be studied based on cell blebs [2]. A number of different types of cells undergo blebbing in response to mechanical perturbations: the hydrostatic pressure could change the cell shape locally, and the hydrodynamic force could work together with the polymerization force to power protrusions [1]. To investigate the process of cell bleb formation, many theoretical models have also been developed [19–21].

In cryopreservation, the blebbing may happen due to the osmotic shock induced by the addition of cryoprotectants (CPAs). In the literature, most of the work focuses on the development of various approaches to minimize the osmotic damage to cells and the time necessary to load CPAs [22–24]; however, few studies focus on the formation and function of cell blebs. To the best of our knowledge, only Hotamisligil et al. in their pioneering work reported that blebs could be induced by CPAs in oocytes [25], but the significance of blebs still needs to be verified for the common cryopreservation process. The hypertonic extracellular environment can cause cell shrinkage, resulting from the water transport across the plasma membrane. However, the formation and development of protrusions on the cell membrane may prevent the very rapid loss of water (the death of cells is related to the water loss [26, 27]). This is an osmoprotective mechanism, existing in many cells, such as kidney cells [28], epithelial and interstitial cells of the renal medulla [29], hypernatremia cells [30] and diabetes cells [31] (the failure of the osmoprotective mechanism can lead to apoptosis [32]). In the presence of CPAs, the osmoprotective mechanism should also exist, and cell blebs may provide some information on how to alleviate the membrane tension driven by osmosis [33]. They may represent a cellular safety protection to reduce the mortality rate of cells [34, 35]. Therefore, it is important to understand how cell blebs form and function, how they are affected by CPAs, and whether there is a relationship between cell blebs and the mortality of cells in the presence of CPAs, and what is the nature of that relationship.

In the presence of CPAs, the osmotic stress may induce not only cell blebs but also autophagy, an evolutionary-conserved mechanism that depends on lysosomes. It is a dynamic degradation process in eukaryotic organisms, associated with survival, differentiation, development, and homeostasis. Autophagy depends upon Atg proteins, which play a central role in integrating signals downstream of the mammalian target of rapamycin (mTOR) [36]. The hyperosmotic environment around mammalian cells results in cell shrinkage with a decrease in water content, and it rapidly (usually less than 5 minutes) and specifically affects a pool of mTOR complexes [37]. mTOR relays the signal and triggers the adaptive changes in the cytoskeleton to withstand physical changes (e.g., cell shrinkage) [38]. Further, Paula Nunes et al. have proved that the hypertonic stress can promote autophagy, which enhances the cell survival and the cytoskeleton remodeling [39]. Both cell blebs and autophagy may be induced by the hypertonic stress and improve the cell survival. We are interested in investigating the relation between cell blebbing and autophagy.

The objective of this study is to determine the protective role of blebs against the hyperosmotic damage to cells in the presence of CPAs and the cryoprotectant concentration dependence of the blebbing. We defined a bleb index correlated with the mortality rate of cells to reflect the pressure a bleb withstands. Using this index, the advantage of the stepwise addition of CPAs in reducing the mortality rate of cells was explained. In addition, the function of induced autophagy was briefly compared with that of cell blebs. Our results presented here contribute to the understanding of the cell tolerance, the reorganization of cytoskeleton and the protective mechanism of blebs in the presence of CPAs. They will help the development of new strategies for CPA loading and will advance the field of cryopreservation.

## Materials and Methods

### Cell Culture

The human cervical carcinoma cell line (HeLa) (Cell bank, Shanghai, China) was cultured continuously at 37°C and 5% CO_2_ in Dulbecco’s modified Eagle's medium (DMEM; Gibco, USA) containing 10% fetal bovine serum (FBS; Gibco, USA) and 1% penicillin-streptomycin (Gibco, USA). Cell culture dishes (MatTek Co., Ashland, MA, USA) were coated with 100 μg/mL of fibronectin (Sigma-Aldrich, USA) at 25°C for 2 hours and then washed 3 times with phosphate buffered saline (PBS) before use. 5000–6000 HeLa cells were seeded in one well of 96-well plates (Costar, Corning Incorporated, New York, USA). After adherence for 12 hours, cells were washed with PBS before the treatment.

### CPA Loading

DMSO and glycerol were purchased from Shangon Company (Shanghai, China). Ten concentrations of CPA solution, 5%, 10%, 15%, 20%… 75% and 80% (v/v), were prepared with PBS. Single-step addition experiments involved the following: DMSO solution (200 μL) containing a particular concentration of CPA was added to a single well. Then, cell blebs and fluorescence were observed with an inverted fluorescence microscope (Olympus American Inc., Center Valley, PA, USA). Stepwise addition experiments involved the following: add 20% of DMSO solution (200 μL) to one well, equilibrate for 30 minutes, replace it with 40% of DMSO solution (200 μL) and equilibrate for 30 minutes. The above steps were repeated until the target concentration was reached, and the cell blebs and fluorescence were observed after each step.

### Cell Viability Assay

Two fluorescent dyes, Hoechst 33258 and Propidium Iodide (PI), were purchased from Sanggon Company and used to detect the mortality of cells. Hoechst 33258 is a blue fluorescent dye used to stain DNA in living or dead cells. Here, a concentration of 1.0 μg/ml Hoechst was used to stain HeLa cells for 1 ~ 30 minutes at room temperature for cell counting [40, 41]. PI is a fluorescent intercalating agent that can bind to DNA. It does not permeate through the membrane of viable cells, and it is commonly used for the identification of dead cells in a population. Hoechst/PI was mixed with CPAs in our experiments.

### Cell Staining

The cytoskeleton was labeled with FITC-phalloidin (Sigma-Aldrich, USA). The extracellular solution was removed with a pipette. Cells were fixed for 10 minutes with a 4% formaldehyde solution in PBS, and washed extensively with PBS. Then, cells were stained with a 10 μg/mL FITC-phalloidin conjugate solution (the solution was prepared with PBS and supplemented with 1% methanol) for 60 minutes at 37°C. Finally, wells were washed several times with PBS to remove unbound phalloidin conjugate, and the cells were observed with an inverted fluorescence microscope. The cell membrane was labeled with DiI (1,1'-dioctadecyl-3,3,3',3'-tetramethylindocarbocyanine perchlorate, Beyotime, Beijing, China). The cytoplasm was labeled with CFSE (carboxyfluorescein diacetate succinimidyl ester Beyotime, Beijing, China). Before staining, the extracellular solution was removed with a pipette. Then, cells were incubated with CFSE solution (10 μM) for 30 minutes or DiI solution (5μM) for 5 minutes at 37°C. Finally, cells were washed with PBS for 3 times for use.

### GFP-LC3 Dots and GFP-Tagged Aggregates

GFP-LC3 dot formation in GFP-LC3/HeLa cells was observed using fluorescence microscopy. Pictures were captured randomly. The GFP puncta were quantified by counting 500 cells and the autophagy rate was expressed as the ratio of the number of cells with at least three GFP-LC3 dots to the number of cells with green fluorescence.

### Western Blot Analysis

Harvested cells were resuspended in the lysis buffer (0.5% Nonide P-40, 10 mM Tris-HCl, pH 7.5, 100 mM NaCl) on ice. One-quarter volume of 5×SDS (Sodium Dodecyl Sulfate) sample loading buffer (the buffer contained 100 mM Tris-HCl, 2% beta-mercaptoethanol, 4% SDS, 20% glycerol and 0.02% bromphenol blue, and its pH value was 6.8) was added, followed by boiling for 10–15 minutes. Proteins separated by SDS/PAGE were transferred to a nitrocellulose transfer membrane (GE Healthcare, USA). The membrane was blocked with 5% nonfat dry milk in TBST (the Tris-buffered saline contained 0.1% Tween-20), incubated overnight at 4°C with a primary antibody at 1:1000 dilution, washed 4 times (8 minutes per wash) with TBST, then incubated with horseradish peroxidase-conjugated secondary antibody (1:10,000 dilution; Promega, Madison, WI, USA) for one hour at room temperature, washed extensively with PBS, and finally visualized by enhanced chemiluminescence (ECL; Thermo Fisher Scientific Inc., Waltham, MA, USA).

### Microscopy Imaging and Processing

An inverted fluorescence microscope fitted with a 40×objective lens and a 100 W mercury lamp (Olympus; 561 nm) was used to observe cell blebs and fluorescence, and the Retiga SRV camera (QImaging, Surrey, BC, Canada) was used for image recording. The actin microfilament was observed with a 63×objective lens by a confocal fluorescence microscope (Zeiss LSM510, Germany). Images were processed using Image J (images were cropped, rotated, and their contrast and brightness were manually adjusted uniformly).

## Results and Discussion

### Cell Blebs Induced by the CPA Addition

**Fig 1:**
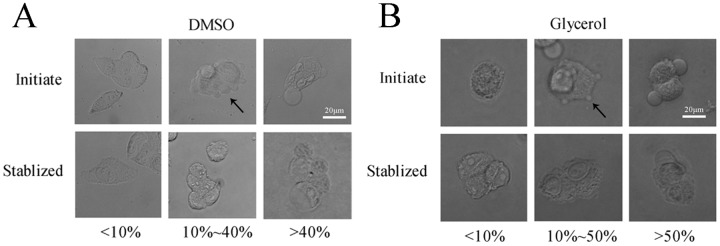
Cell blebs induced by the addition of CPAs. Various concentrations of **(A)** DMSO and **(B)** glycerol were applied to HeLa cells for 30 minutes. The development of cell blebs during the first 3 minutes was observed as the initial state and after 30 minutes as the stable state. Initiate: 3 minutes, and Stabilized: 30 minutes. The experiments were repeated 3 times.

**Fig 2:**
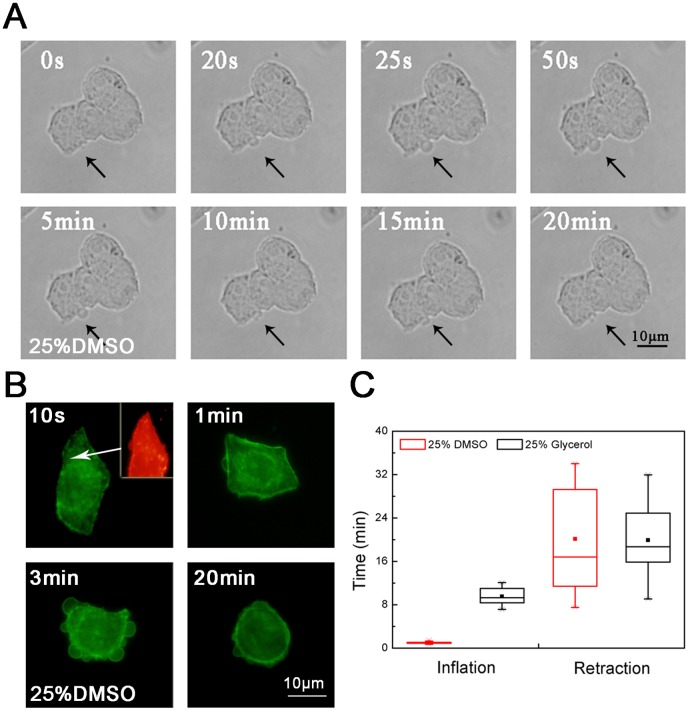
Life cycle of a dynamic bleb. **(A)** the inflation and retraction of one bleb (black arrows); **(B)** the actin microfilament reorganization during the bleb inflation and retraction; **(C)** the comparison of the inflation and retraction time between DMSO and glycerol. For **(A)** and **(B)**, the experiments were repeated 3 times. For **(C)**, the number of cells used was approximately 20.

The inverted fluorescence microscope was used to observe the formation and development of cell blebs after the addition of DMSO or glycerol. From the initial stimulation to the stable state, cell blebs are generated in different shapes and sizes. For the DMSO concentrations above 40% (v/v), cells may form some large stationary blebs (Fig 1A). In contrast, cells incubated with the DMSO concentrations below 10% form only a few blebs. Compared to DMSO, glycerol is milder in stimulating the formation of cell blebs and dynamic blebs can appear between 10% and 50% (Fig 1B). Cell blebs induced by the addition of CPAs inflate first and then retract (Fig 2A). In the inflation of blebs, a cortical cytoskeleton starts to assemble underneath the bleb membrane (Fig 2B). In the retraction of blebs, the contractile cortex drives the extruded cytoplasm back into the cell body [1]. For different cryoprotectant types, the inflation and retraction time of blebs is different. In this study, the inflation time was ~ 1 minute for DMSO whereas ~ 10 minutes for glycerol, and the difference of the retraction time was not significant (Fig 2C). The results here confirm that upon the addition of CPAs, the hypertonic stress can induce the formation of blebs on the cell membrane. We note that the large stationary blebs are sometimes mentioned in the literature in the context of apoptosis or necrosis. Although some apoptosis blebs can also shrink, they cannot be quantified as spherical blebs. They quickly lose their symmetry. In addition, the retraction of these blebs is not complete, and most blebs eventually become small irregular remnants [42].

### Mechanism of Cell Bleb Formation

**Fig 3:**
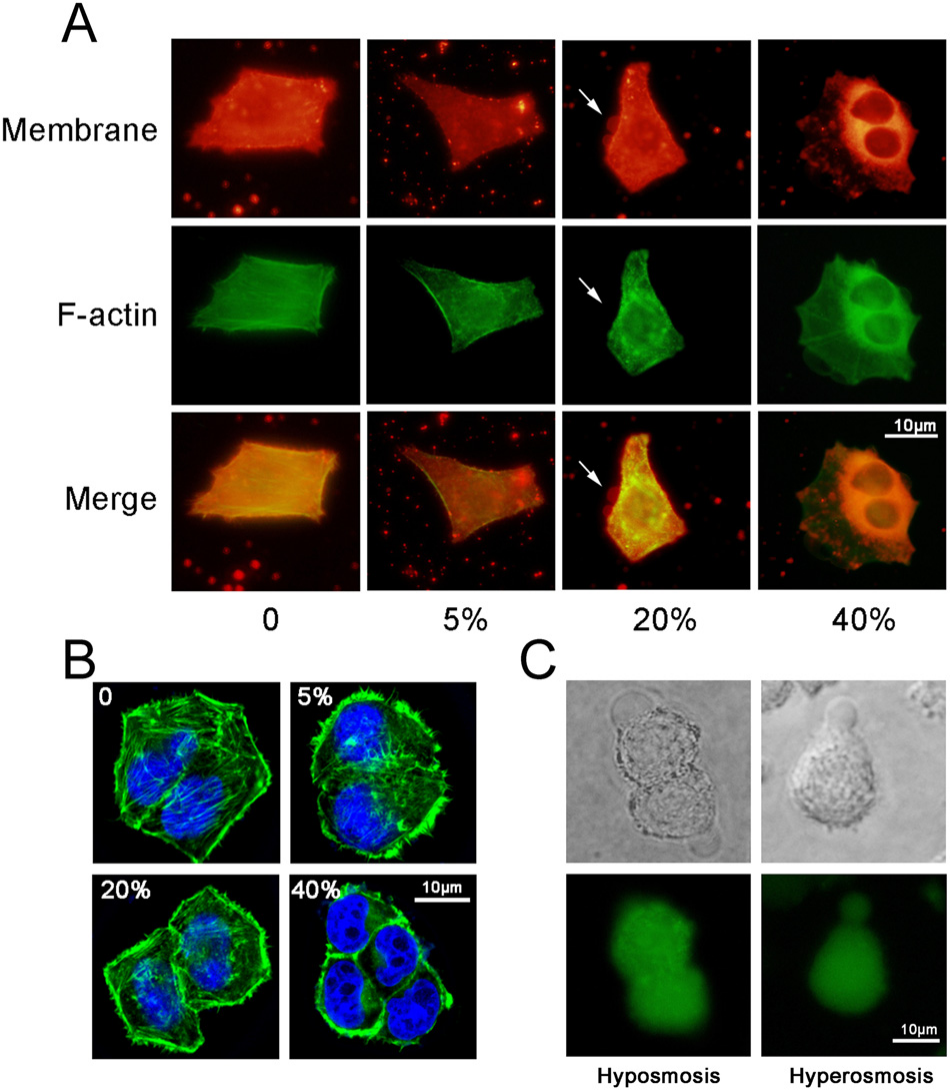
Cell blebs and cytoskeleton under different DMSO concentrations. **(A)** The bleb and cytoskeleton were observed by an inverted fluorescence microscope (membrane: red; cytoskeleton: green). **(B)** The bleb and cytoskeleton were observed by a confocal microscope (cytoskeleton: green; nucleus: blue). **(C)** The fluid flows in the formation of blebs under a hypoosmotic condition (0.1×PBS) and a hyperosmotic condition (25% DMSO in PBS). The experiments were repeated 3 times.

To observe the changes in the cytoskeleton and the cell membrane upon the addition of cryoprotectants, FITC-phalloidine (green fluorescence) and DiI (red fluorescence) were used to label the cytoskeleton (the F-actin) and the cell membrane, respectively. DMSO at concentrations of 5%, 20% and 40% (v/v) were introduced in a single-step addition mode. After 10 seconds, cells were fixed and stained for actin analysis. In a control group (PBS treatment only), the cell membrane is attached to the cortex. Cells exposed to 5% DMSO display no significant detachment of the membrane from the cortical cytoskeleton (Fig 3A) but a disassembled actin microfilament structure (Fig 3B; the cortical cytoskeleton was discontinuous with patchy intense areas of actin staining). However, in cells exposed to 20% DMSO, the detachment of the cell membrane from the cortical cytoskeleton occurs. In addition, in cells exposed to 40% DMSO, the actin microfilament organization disappears completely and the actin microfilaments aggregate around the chromosomes. The results here show the actin microfilament organization was slightly affected by exposure to less than 20% DMSO, were damaged but not destroyed by exposures between 20% and 40%, and were disrupted by exposure to more than 40%.

The extracellular hyperosmotic stress upon the addition of cryoprotectants causes the loss of the intracellular water and thus, the shrinkage of cells. The shrinkage of cells creates a compressive stress on the cytoskeletal network, resulting in not only the localized cytoskeleton damage (Fig 3B) but also the increase in the intracellular hydrostatic pressure that can lead to the localized detachment of the plasma membrane from the underlying cytoskeleton in the region where the membrane-cortex attachment is weak. The detached membrane rapidly inflates as the cytoplasm flows in and then the bleb forms. In this study, we thought that the blebbing is driven primarily by the cytoplasm flow within the cell rather than water across the plasma membrane. To verify the speculation, experiments were conducted to compare two kinds of blebs formed under a hypoosmotic condition (0.1×PBS) and a hyperosmotic condition (25% DMSO in PBS), respectively. In experiments, CFSE (green fluorescence) was used to label the total protein in cytoplasm. The results show that when the hypoosmotic solution was added, the blebbing was driven by the water outside cells as no protein appeared in the bleb (Fig 3C). However, when the hyperosmotic solution was added, the blebbing was driven by the flow of cytoplasm as the cytoplasm appeared in the bleb. Therefore, the blebbing induced by the addition of cryoprotectants is driven primarily by the intracellular fluid flow. The conclusion here is consistent with that in the literature [1].

### Function of Cell Blebs in the Presence of CPAs

**Fig 4:**
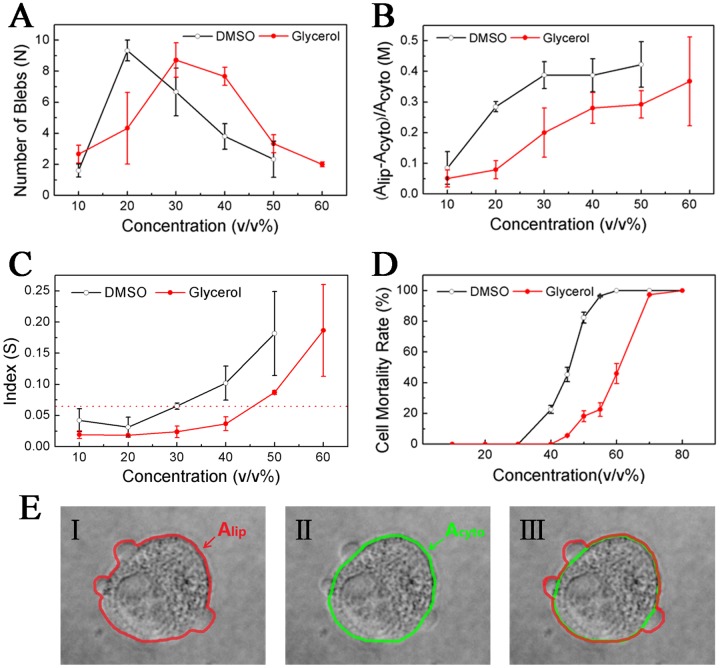
Effect of the concentration of CPAs: (A) number of cell blebs; (B) total area of cell blebs; (C) bleb index; (D) mortality rate of cells; (E) schematic of A_lip_ and A_cyto_. HeLa cells were treated with a series of solutions containing different amounts of DMSO or glycerol, as well as the fluorochromes Hoechst and PI. After 30 minutes, when cells were stable, an inverted fluorescence microscope was used to observe cell death. For **(A)**, **(B)** and **(C)**, the cell number was approximately 40. For **(D)**, the cell number was approximately 500 and the experiment was repeated 5 times. For **(E)**, the red boundary denotes the lipid bilayer and the green boundary denotes the cortical cytoskeleton.

To determine the role of blebs and the relationship between the blebs and the mortality rate of cells, we defined a bleb index *S*: *S* = *M* / *N* = (*A*_*lip*_—*A*_*cyto*_) / *A*_*cyto*_ / *N* = *A*_*bleb*_ / *A*_*cyto*_. *N* is the number of blebs, *A*_*lip*_ is the 2D area surrounded by the lipid bilayer (Fig 4E, red boundary), *A*_*cyto*_ is the 2D area occupied by the cortical cytoskeleton (Fig 4E, green boundary), and *A*_*bleb*_ is the averaged area of blebs. To establish a quantitative relation between *S* and osmotic/hydrostatic pressure, we followed the pioneer work in the literature [16]. We assumed that the change in the cell radius after blebbing is neglectable (in fact, since cells flatly stuck to the surface of dishes in this study, the cortex area *A*_*cyto*_ or the cell radius *r*_*c*_ could be considered constant). Then, the approximate equation *T*/*r*_*c*_ = *γ*/*r*_*b*_ can be obtained (*T* = *γ* + *ξh*/2 is the total cell tension, *γ* is the membrane tension, *h* is the cortex thickness, *ξ* is the active stress exerted by myosin, and *r*_*b*_ is the bleb radius). Because at equilibrium *P*_*c*_ = *P*_*b*_ and Δ*π* = *π*_*c*_ − *π*_*ε*_ = Δ*P* = *P*_*c*_ − *P*_*ε*_, one finds Δ*π*(1 − *r*_*b*_/*r*_*c*_) = ξ*h*/*r*_*c*_ (*P*_*b*_ = *P*_*e*_ + 2*γ*/*r*_*b*_ is the hydrostatic pressure in the bleb, *π*_*c*_ and *π*_*e*_ are intracellular and extracellular osmotic pressures, *P*_*c*_ and *P*_*e*_ are intracellular and extracellular hydrostatic pressures, Δ*π* and Δ*P* are transmembrane osmotic and hydrostatic pressure differences). When the bleb radius is far less than the cell radius, one can get $\Delta \pi \approx \left(\xi h/r_{c}\right)\left(r_{b}/r_{c}\right)\approx \left(\xi h/r_{c}\right)\sqrt{A_{bleb}/A_{cyto}}=\xi h\sqrt{S}/r_{c}$. Hence, the transmembrane osmotic/hydrostatic pressure difference or the change in the pressure difference after blebbing is in direct proportion to $\sqrt{S}$. The relation also indicates that S can reflect the hydrostatic pressure in cells. It should be noted that in this study, cell blebs occurred not only at the cell edge but also on the cell top; consequently, it is very difficult to develop an accurate quantitative equation to describe the relation between the index and osmotic/hydrostatic pressure.

In the presence of CPAs, the induced bleb rapidly expands as the cytoplasm flows in. The cytoplasm flow is mainly driven by the hydrostatic pressure and opposed by the membrane tension. The hydrostatic pressure will drop as the bleb expends. The localized bleb can be considered as a cell self-protective response to avoid the global cell damage. Our results show that as the CPA concentration increases, *N* increases initially and then decreases (Fig 4A) whereas *M* and *S* increase monotonically (Fig 4B and 4C, the software Image J was used to calculate these 2D areas by counting pixels). In this study, the maximum number of blebs appears at approximately 20% DMSO or 30% glycerin (Fig 4A), and the bleb index S = 0.065 can be regarded as a reference value for the safe addition of DMSO to HeLa cells by comparing the bleb index in Fig 4C with the cell mortality rate in Fig 4D. For the safe addition of glycerin, the reference index is 0.037. With less than 30% DMSO (or 40% glycerin), the formation of dynamic blebs and the mortality rate of cells are extremely low. However, in the presence of CPAs at higher concentrations, the extreme hyperosmotic stress destroys rapidly the cytoskeleton and the connection between the membrane and the cortex, injuring the cell. Therefore, the formation of dynamic blebs benefits the cell survival. In summary, upon the addition of CPAs, cells release the pressure inside cells by forming multiple blebs, or they avoid a collapse by rearranging the microfilament network (cells keep the basic structural and functional integrity by sacrificing the local cortex). In other words, the intracellular hydrostatic pressure induced by the extracellular hyperosmotic shock results in the local rupture of the cortex and thus, blebs are formed. Multiple blebs decrease the induced pressure and thus reduce the cell mortality rate.

### Bleb Index upon the Stepwise Addition of CPAs

**Fig 5:**
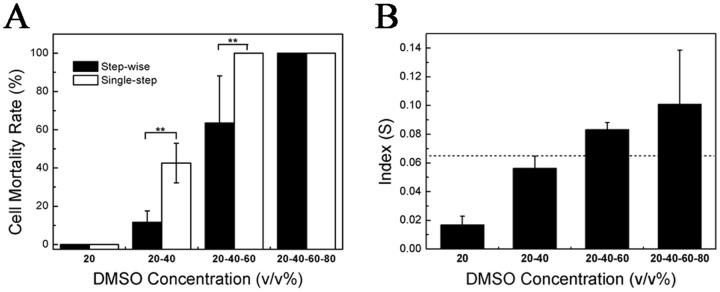
Stepwise addition of DMSO. **(A)** Comparison of the mortality rate of cells between stepwise and single-step addition. **(B)** Bleb index in the stepwise addition method. HeLa cells were treated with 20% DMSO for 30 minutes, and the solution was removed quickly and changed to 40% DMSO for 30 minutes. It was then changed to 60% DMSO for 30 minutes and, finally, to 80% DMSO. The inverted fluorescence microscope was used to observe dead cells labeled by PI and Hoechst. For **(A)**, the number of cells used was approximately 500 and the experiment was repeated 5 times. For **(B)**, the number of cells used was approximately 40. **p<0.01 was considered statistically significant.

The stepwise addition of CPAs has been widely used in cryopreservation. Having established that cell blebs are essential for the hyperosmotic stress relaxation upon the CPA addition, we wonder whether the bleb index can be used to explain the advantage of the stepwise addition of CPAs. To verify our hypothesis, we added DMSO in a stepwise mode (the concentration increment was 20%, and the target concentration was 80%). At each step, cells were incubated for 30 minutes, and PI and Hoechst were then used to detect cell death. Our results show that in the stepwise addition method, the mortality rate of cells increases with the number of steps (the higher the concentration of DMSO is, the more the number of steps is). However, it is always less than the rate in the single-step method (Fig 5A). In the stepwise addition method, the bleb index also increases with the number of steps (Fig 5B), but is far less than the one in the single-step method. For example, the index of two steps to 40% was 0.056 whereas the index of one-step to 40% was 0.102 (Fig 4C). In the stepwise addition, for each step, the hydrostatic pressure induced by the hypertonic shock is reduced and thus the pressure a bleb withstands (the size of blebs) is reduced. However, due to the incomplete retraction of the bleb in the previous step, the bleb size is accumulated to the next step and thus, the bleb index increases with the step number. In the stepwise addition, although the accumulated size of blebs is also large, it is still far less than the size in the single-step addition. Therefore, the small bleb index can explain the advantage of the stepwise addition of CPAs.

### Cell Blebs vs. Autophagy

**Fig 6:**
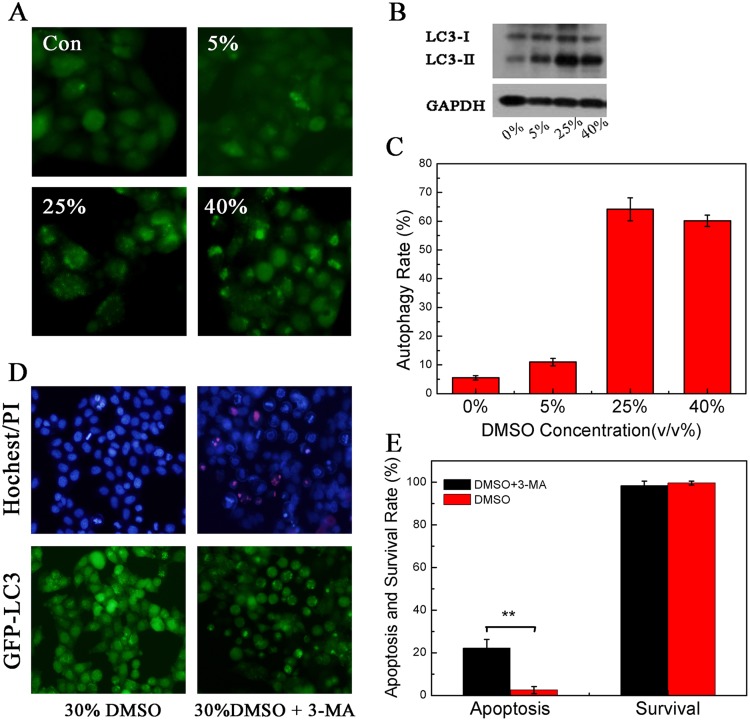
The autophagy induced by the addition of CPAs. **(A)** GFP-LC3/HeLa cells were treated with various concentrations of DMSO, and GFP green fluorescence dots appeared in cells. **(B)** LC3 conversion was determined by western blot in HeLa cells treated with different concentrations of DMSO. **(C)** Effect of DMSO on the autophagy rate. **(D)** GFP-LC3 /HeLa cells were inhibited by 3-MA, and then stimulated by 30% DMSO. Shrinkage of cell nuclei is a hallmark of apoptosis. **(E)** Autophagy reduced the apoptosis in the presence of 30% DMSO. **p<0.01 was considered statistically significant. The experiments were repeated 5 times. The number of cells used was approximately 500.

Upon the addition of CPAs, blebbing may not be the only factor that can protect cells because the hypertonic stress may cause autophagy and thus improve the cell survival [39]. To investigate the relation between blebbing and autophagy, we studied autophagy of cells in the presence of CPAs. In this work, we used Atg8/LC3-I as an autophagic marker to evaluate autophagy intensity [43]. Autophagy involves the generation of membrane-binding LC3-II via fusion between phosphatidyl-ethanolamine (PE) and LC3-I. This complex subsequently connects to the outer and inner membranes of the autophagosome [44]. We treated GFP-LC3/HeLa cells with different concentrations of DMSO. GFP-LC3/HeLa cells stably expressed GFP-LC3 exogenous fusion proteins (GFP: green fluorescent protein; LC3: microtubule-associated light chain 3 protein) [45]. The cells were treated for 30 minutes to observe the formation of GFP puncta. We used western blot assay to analyze the conversion from LC3-I to LC3-II. Our results show that the formation of GFP puncta can be upregulated by DMSO in GFP-LC3/HeLa cells in a dose-dependent manner. No such puncta formed with low concentrations of DMSO (Fig 6A and 6C). DMSO triggers the conversion from LC3-I to LC3-II and increases the relative LC3-II/ GAPDH ratio (Fig 6B). Therefore, the hypertonic stress affects the cytoskeleton reorganization and thus induces cell autophagy. In this work, 3-methyladenine (3-MA), which can inhibit cell autophagy by blocking autophagosome formation [46], was used to study the mechanism of autophagy under the hypertonic condition 3-MA. We investigated the mortality rate of cells following the addition of 30% DMSO after the treatment with 3-MA for 12 hours. Our results show that the mortality rate of cells is not increased; however, the nuclear shrinkage, a typical feature of apoptosis, appears when autophagy is inhibited (Fig 6D and 6E). Thus, autophagy is also a self-protective mechanism for avoiding cell death by reducing cell apoptosis (in Fig 6D, GFP puncta are still generated in some cells, possibly resulting from an incomplete inhibition). In this work, we distinguished whether the apoptosis occurs according to the shape of the nuclei of cells. Normally, the nucleus of cells is round. However, once the cells begin apoptosis, they will exhibit nuclear condensation.

In the presence of CPAs, the hypertonic stress results in the breakage of the cortex, causing blebbing and autophagy, which can both enhance cell survival. However, the corresponding mechanisms of cell protection are different in the two cases: blebbing directly releases the stress, whereas autophagy indirectly inhibits the apoptosis. The first mechanism is faster than the second is.

## Conclusions

In cryopreservation, the effect of the volume change of cells upon the addition of CPAs on the recovery rate of cells has been studied widely. However, the cryoprotectant- induced bleb on the cell membrane during the process has not been deeply investigated and thus the formation mechanism and function of blebs remain unclear. In addition, because of not considering the presence of blebs, the reliability of the lower volume tolerance limit established for the safe addition of CPAs according to the relationship between the cell volume and the cell mortality to some extent is questionable. Therefore, it is of significance to study the blebs induced by the addition of CPAs and further define a bleb-based parameter for the safe addition of CPAs.

In this study, the formation and mechanism of blebs upon the addition of CPAs was investigated. The results show the formation of blebs results from the hypertonic-induced compressive stress on the cytoskeletal network, followed by the rupture of the cell membrane from the underlying cytoskeleton. The actin microfilaments are almost intact when cells are exposed to less than 10% DMSO, are damaged but not destroyed when exposed to between 20% and 40% DMSO, and are disrupted when exposed to more than 40% DMSO. In practice, the blebbing protects cells against hypertonic damage, but the effect is pronounced only when the concentration of CPAs is low. In other words, within a certain concentration range of CPAs, the cortex of the cytoskeleton is only locally damaged rather than completely collapsed. Therefore, dynamic blebs form, and they can protect cells because actin filaments can be reconstructed.

The formation of blebs is a self-protecting mechanism against the osmotic damage to cells: multiple blebs decrease the intracellular hydrostatic pressure induced by the extracellular hyperosmotic shock and thus decrease or even prevent a sudden and complete collapse of the cortical cytoskeleton, reducing the mortality rate of cells. In this work, we defined a bleb index S correlated with the mortality rate of cells to reflect the pressure a bleb withstands. We found that the bleb index S = 0.065 may be considered a reference for the safe addition of DMSO to HeLa cells. Corresponding to the bleb index below 0.065, when the DMSO concentration is below 30%, the cell mortality rate is very low. In addition, the bleb index can also explain why the stepwise addition of CPAs is better than the single-step addition of CPAs.

In the presence of CPAs, the osmotic stress change can also induce autophagy. Although autophagy may improve cell survival, the associated mechanism is different from that of the blebbing. The blebbing is possibly a primary self-protection process because it can rapidly release the stress inside cells and achieve an immediate effect.
